# Plasma procalcitonin is associated with all-cause and cancer mortality in apparently healthy men: a prospective population-based study

**DOI:** 10.1186/1741-7015-11-180

**Published:** 2013-08-13

**Authors:** Ovidiu S Cotoi, Jonas Manjer, Bo Hedblad, Gunnar Engström, Olle Melander, Alexandru Schiopu

**Affiliations:** 1 Department of Cellular and Molecular Biology, University of Medicine and Pharmacy of Tîrgu Mureş, Tîrgu Mureş, Romania; 2 Department of Clinical Sciences, Lund University, Lund, Sweden; 3 Cardiology Clinic, Skane University Hospital, Malmö, Sweden

**Keywords:** Procalcitonin, Inflammation, Cancer, Cardiovascular disease, CRP, Mortality

## Abstract

**Background:**

The inflammatory mediator procalcitonin (PCT) has previously been associated with prognosis in myocardial infarction, cancer and sepsis patients. The importance of PCT in the general population is currently unknown. Our aim was to assess the relationship between plasma PCT and the risk of all-cause and cause-specific mortality in apparently healthy individuals with no previous history of cardiovascular disease or cancer.

**Methods:**

We performed a prospective, population-based study on 3,322 individuals recruited from the Malmö Diet and Cancer cohort, with a median follow-up time of 16.2 years. Plasma PCT, high-sensitivity C-reactive protein (hsCRP), low-density lipoprotein (LDL), high-density lipoprotein (HDL), triglycerides and cystatin C were measured at baseline and a thorough risk factor assessment was performed for all subjects. The primary end-points of the study were all-cause mortality, cancer mortality and cardiovascular mortality.

**Results:**

Men had higher PCT levels compared to women. In Cox proportional hazard models adjusted for age, sex, hypertension, diabetes, plasma lipids, renal function, body mass index and smoking, baseline PCT was associated with all-cause mortality and cancer mortality in men. The hazard ratio (HR) for men with PCT levels within the highest compared with the lowest quartile was 1.52 (95% confidence interval (CI) 1.07 to 2.16; *P* = 0.024) for all-cause mortality and 2.37 (95% CI 1.36 to 4.14; *P* = 0.006) for cancer mortality. Additionally, men with increased plasma PCT were found to be at a higher risk to develop colon cancer (HR per 1 SD increase = 1.49 (95% CI 1.13 to 1.95); *P* = 0.005). In multivariate Cox regression analyses with mutual adjustments for PCT and hsCRP, PCT was independently associated with cancer death (HR per 1 SD increase = 1.28 (95% CI 1.10 to 1.49); *P* = 0.001) and hsCRP with cardiovascular death (HR per 1 SD increase = 1.42 (95% CI 1.11 to 1.83); *P* = 0.006) in men. We found no significant correlations between baseline PCT or hsCRP and incident cancer or cardiovascular death in women.

**Conclusions:**

We disclose for the first time important independent associations between PCT and the risk for all-cause and cancer mortality in apparently healthy men. Our findings warrant further investigation into the mechanisms underlying the relationship between PCT and cancer.

## Background

Inflammation plays a central role in the pathogenesis of cardiovascular (CV) disease [1] and cancer [2], the two most common causes of death worldwide among non-communicable diseases [3]. The inflammatory biomarker high sensitivity C-reactive protein (hsCRP) correlates with CV morbidity and mortality in the general population and has been shown to mildly improve CV risk prediction on top of the Framingham Risk Score [4]. Numerous immune and inflammatory conditions have been linked to increased risk of cancer development [5]. Plasma hsCRP has been shown to predict the incidence of certain types of cancer [6] and has been linked to the risk of all-cause and cancer death [7,8].

Procalcitonin (PCT) is the precursor of the hormone calcitonin which functions as a regulator of calcium metabolism. PCT is the product of the *Calc1* gene and is secreted under homeostatic conditions by the neuroendocrine cells of the thyroid gland and lungs [9]. PCT concentrations are low in healthy individuals but increase dramatically in the systemic inflammatory response syndrome (SIRS) associated with severe infections and sepsis [10] as well as with trauma, surgery, burns and pancreatitis [11]. The levels of PCT reflect disease severity and predict resolution and prognosis [10,12,13]. Under these conditions it has been suggested that the entire body may become a source of PCT, as *Calc1* mRNA was found to be expressed in almost all tissues examined in animal models of sepsis [14,15]. PCT was shown to have pro-inflammatory and immunosuppressive properties and to be actively involved as a mediator of disease progression and severity in sepsis [15-17].

The importance of PCT under homeostatic conditions in the general population has been studied to a much lesser extent. Due to the importance of PCT as an immune and inflammatory mediator we hypothesized that PCT might be involved in pathologic conditions other than SIRS and sepsis. We have recently demonstrated a positive association between the levels of plasma PCT in an unselected population of apparently healthy individuals and the incidence of coronary events and CV death [18]. However, PCT did not provide supplementary information for CV risk prediction due to a high degree of co-variation with hsCRP and traditional CV risk factors, such as diabetes and blood pressure [18].

Here we investigate whether plasma PCT in apparently healthy individuals with no previous history of cancer or CV disease is associated with total and cause-specific mortality. Additionally, we assessed whether there are gender-specific differences with regard to the value of PCT as a mortality predictor, as men have generally higher baseline PCT levels than women [18]. PCT was analyzed alone and in comparison with hsCRP. We demonstrate that PCT is strongly associated with the risk of all-cause and cancer mortality and with the incidence of colon cancer in men, independently of smoking, diabetes, hypertension, body mass index (BMI), plasma lipids, renal function and hsCRP. In contrast, hsCRP was independently associated with CV mortality but not with cancer mortality in men. Neither biomarker was correlated with incident mortality in women, after adjustment for potential confounding risk factors.

## Methods

### Study design and population

The study population was part of the CV arm of the Malmö Diet and Cancer (MDC) cohort [19]. The MDC is a population-based prospective cohort of 28,449 individuals enrolled between 1991 and 1996. The CV arm of the MDC includes 6,094 subjects selected for the study of CV disease [20]. Out of this cohort we excluded 89 individuals who had previously been diagnosed with coronary disease or stroke and an additional 619 subjects who had a previous cancer diagnosis. Of the remaining individuals, overnight fasting plasma samples for PCT measurement were available for 3,322 subjects. The characteristics of the included and excluded subjects are presented in Table 1 and Additional file 1: Table S1. None of the participants had clinical signs of infection upon enrollment and hsCRP values were within the normal range (Table 1). All participants provided written informed consent and the study was approved by the ethical committee at Lund University, Sweden and conducted in accordance with the Helsinki declaration.

**Table 1:** Characteristics of the study population at baseline

**Characteristic**	**Population**	**Men**	**Women**	**PCT quartiles**			
				**Q1**	**Q2**	**Q3**	**Q4**
Number of participants (%)	3,322	1,467 (44)	1,855 (56)	935	820	760	807
Age, mean (SD), years	58 (6)	58 (6)	58 (6)	56 (6)	57 (6)	58 (6)	59 (6)
Blood pressure, mean (SD), mm Hg							
Systolic	142 (19)	143 (18)	140 (19)	137 (18)	140 (18)	145 (19)	146 (19)
Diastolic	87 (9)	89 (9)	86 (9)	85 (9)	86 (9)	88 (9)	89 (9)
Hypertension, Number (%)	2,118 (63.8)	991 (67.6)	1,127 (60.8)	508 (54.3)	506 (61.7)	510 (67.1)	594 (73.6)
Lipids, mean (SD), mmol/L							
LDL-C	4.17 (0.98)	4.12 (0.90)	4.20 (1.04)	4.05 (1.00)	4.11 (0.94)	4.30 (0.99)	4.22 (0.97)
HDL-C	1.38 (0.37)	1.22 (0.29)	1.52 (0.37)	1.52 (0.38)	1.41 (0.36)	1.32 (0.35)	1.26 (0.33)
TG	1.31 (0.63)	1.43 (0.68)	1.21 (0.57)	1.11 (1.00)	1.22 (0.55)	1.40 (0.66)	1.53 (0.73)
Body mass index, mean (SD), kg/m^2^	25.7 (3.9)	26.0 (3.4)	25.5 (4.2)	24.9 (3.7)	25.3 (3.7)	26.1 (3.7)	26.8 (4.0)
Diabetes mellitus, Number (%)	272 (8.2)	158 (10.8)	114 (6.1)	42 (4.5)	41 (5.0)	71 (9.3)	118 (14.6)
Current smoking, Number (%)	861 (25.9)	412 (28.1)	449 (24.2)	251 (26.8)	221 (27.0)	205 (27.0)	184 (22.8)
Cystatin C, mean (SD), mg/L	0.77 (0.14)	0.79 (0.15)	0.75 (0.13)	0.72 (0.12)	0.76 (0.12)	0.79 (0.13)	0.83 (0.17)
hsCRP, median (IQR), mg/L	0.13	0.12	0.13	0.10	0.11	0.13	0.20
	(0.06 to 0.28)	(0.06 to 0.27)	(0.06 to 0.28)	(0.05 to 0.20)	(0.06 to 0.24)	(0.07 to 0.27)	(0.09 to 0.41)
PCT, median (IQR), pg/mL	16 (13 to 20)	18 (15 to 23)	14 (12 to 18)	12 (10 to 13)	15 (14 to 16)	18 (17 to 19)	25 (22 to 30)
Men				14 (12 to 15)	17 (16 to 18)	20 (19 to 21)	28 (25 to 34)
Women				10 (9 to 11)	13 (12 to 14)	16 (15 to 17)	21 (19 to 25)

hsCRP, high sensitivity C-reactive protein; HDL-C, high-density lipoprotein cholesterol; IQR, interquartile range; LDL-C, low-density lipoprotein cholesterol; PCT, procalcitonin; TG, triglycerides.

### Data collection

All participants provided a medical history and underwent a physical examination. Cigarette smoking was assessed by a self-administered questionnaire and was defined as any smoking within the past year. Blood pressure was measured after resting for 10 minutes in the supine position. Hypertension was defined as systolic blood pressure (SBP) ≥140 mmHg, diastolic blood pressure (DBP) ≥90 mmHg or use of antihypertensive medication. Diabetes mellitus was defined as a fasting whole-blood glucose level >6.0 mmmol/L, a self-reported physician diagnosis of diabetes or use of antidiabetic medication. Plasma lipids were measured at the Department of Clinical Chemistry, Skane University Hospital. CRP concentration was determined using a high-sensitivity assay (Tina-Quant CRP, Roche Diagnostics, Basel, Switzerland). Cystatin C was measured using a particle-enhanced immunonephelometric assay (N Latex Cystatin C, Dade Behring, Deerfield, IL, USA). Serum PCT concentration was determined by an ultrasensitive assay (ProCa-S; BRAHMS GmbH, Hennigsdorf, Germany). The lower detection limit of the assay was 10 pg/mL and the functional assay sensitivity (the lowest value with an inter-assay coefficient of variation below 20%) was 17 pg/mL, as reported by the assay manufacturer.

### Study endpoints

We studied total mortality and mortality due to CV, malignant, infectious, endocrine, neurological, psychiatric, respiratory, digestive, urogenital, musculoskeletal and dermatological diseases. Secondary endpoints were total cancer incidence, as well as the incidence of colon, rectum, lung and bronchi, breast, prostate, urinary tract and skin cancers. In order to ensure adequate power, only cancer diagnoses with more than 20 incident events during follow-up were included in the analysis. Events were identified through linkage of the 10-digit personal identification number of each Swedish citizen with three registries: the Swedish Hospital Discharge Register, the Swedish Cause of Death Register and the Swedish Cancer Registry (SCR). Classification of outcomes using these registries has previously been validated [21], and the sensitivity of the registry for detecting events such as myocardial infarction has been shown to exceed 90% [22]. Approximately 99% of all tumors diagnosed at Swedish Hospitals are registered in the SCR and 98% are morphologically verified [23]. Tumor site was registered according to the International Classification of Diseases, revision 7 (ICD-7) and the ICD version used at diagnosis. Pathological type was coded according to the C24 classification [23]. CV death was defined using codes 390 to 459 (IDC9) and I codes (ICD10) as the main cause of death in the cause of death registry.

### Statistical analysis

SPSS software (version 19, SPSS Inc, Chicago, IL, USA) was used for all statistical calculations. The values for PCT and hsCRP were logarithmically transformed before being included in the analysis. We performed multivariate Cox proportional hazards analyses to assess the associations between baseline PCT and the risk of all-cause mortality, cancer mortality, CV mortality and combined mortality by other causes. We used two different models adjusted for: (1) age and sex and (2) age, sex, and other risk factors previously associated with morbidity and mortality in the general population (hypertension, BMI, HDL, LDL, TG, diabetes mellitus and smoking) as well as cystatin C as a surrogate marker of renal function, with and without further adjustment for hsCRP. Data were expressed as hazard ratios (HR) and 95% confidence intervals (CI). Further, we tested whether PCT is related to the incidence of different types of cancer using a Cox proportional hazards model adjusted for age and sex. A two-sided value of *P* < 0.05 was considered statistically significant. Receiver operating characteristic (ROC) analyses were used to test the value of PCT as a potentially useful morbidity and mortality predictor.

## Results

### PCT and mortality

We investigated whether increased plasma PCT levels in apparently healthy individuals with no previous history of CV disease or cancer are associated with higher mortality risk. During a median follow-up period of 16.2 years (IQR 15.6 to 16.8), 434 (13%) of the study subjects died. Cancer was the most common single cause of death in the study population (201 cases), followed by CV disease (121 cases) (Table 2). Injuries and poison (21), respiratory (20), neurological (20), digestive (12), psychiatric (9), endocrine, metabolic and immune (6), infectious (4), urogenital (2), dermatological (2) and musculoskeletal (1) diseases accounted for 97 deaths. The cause of death was unknown for 14 individuals. Due to the low number of deaths of each separate cause, deaths due to causes other than cancer and CV disease were analyzed together. Men had higher mortality compared to women (Table 2). Risk factor prevalence in the population by PCT quartile is presented in Table 1 and by cause of death in Additional file 1: Table S2.

**Table 2:** Correlation between PCT quartiles and mortality adjusted for age and sex

**Subjects**	**Outcome**	**Number of events**^**a**^	**Q2 versus Q1**	**Q3 versus Q1**	**Q4 versus Q1**	***P *for linear trend**
			**HR (95% CI)**	**HR (95% CI)**	**HR (95% CI)**	
All subjects	All-cause mortality	8.39	1.05 (0.77 to 1.43)	1.29 (0.96 to 1.73)	1.63 (1.22 –to 2.17)	<0.001***
	Cancer mortality	3.88	1.07 (0.69 to 1.68)	1.47 (0.96 to 1.25)	1.60 (1.04 to 2.43)	0.014*
	CVD mortality	2.34	1.24 (0.67 to 2.29)	1.30 (0.71 to 2.36)	1.94 (1.09 to 3.42)	0.016*
	Mortality from causes other than cancer and CVD	2.16	0.92 (0.50 to 1.68)	1.08 (0.60 to 1.93)	1.49 (0.86 to 2.60)	0.096
Men	All-cause mortality	11.14	0.98 (0.67 to 1.45)	0.98 (0.68 to 1.42)	1.50 (1.08 to 2.09)	0.020*
	Cancer mortality	4.92	1.54 (0.86 to 2.75)	1.23 (0.68 to 2.21)	1.96 (1.15 to 3.35)	0.027*
	CVD mortality	3.13	0.74 (0.34 to 1.61)	0.86 (0.43 to 1.72)	1.41 (0.76 to 2.60)	0.208
	Mortality from causes other than cancer and CVD	3.09	0.65 (0.03 to 1.40)	0.86 (0.44 to 1.67)	1.15 (0.62 to 2.12)	0.557
Women	All-cause mortality	6.30	1.45 (0.86 to 2.46)	1.57 (0.91 to 2.71)	2.04 (1.22 to 2.34)	0.004*
	Cancer mortality	3.13	1.51 (0.77 to 2.99)	1.25 (0.59 to 2.62)	1.62 (0.81 to 3.23)	0.295
	CVD mortality	1.74	1.18 (0.36 to 3.86)	1.91 (0.61 to 6.00)	2.73 (0.93 to 8.06)	0.012*
	Mortality from causes other than cancer and CVD	1.46	1.66 (0.52 to 5.25)	2.17 (0.68 to 6.96)	2.40 (0.78 to 7.38)	0.106

Cox regression analysis adjusted for age and sex. ^**a**^Expressed per 1,000 person years. CI, confidence interval; CVD, cardiovascular disease; HR, hazard ratio; PCT, procalcitonin; Q, quartile. *P <0.05, ***P <0.001.

**Figure 1:**
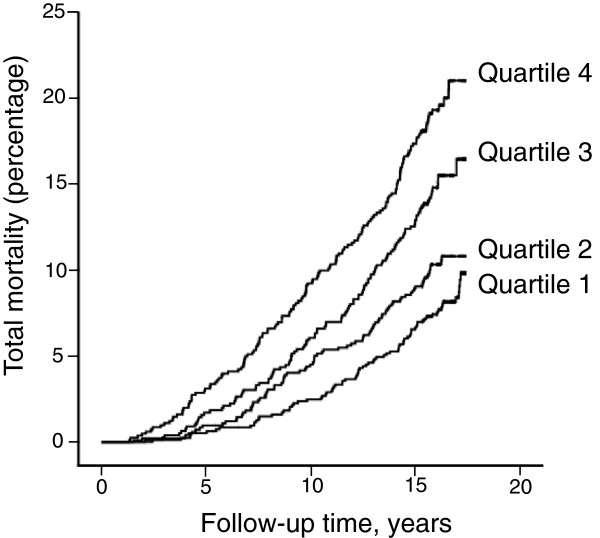
**Total mortality in the study population during follow-up by baseline PCT quartile.** Kaplan – Meier plot showing 1 minus total survival by PCT quartiles: first (lowest values) to fourth quartile of the baseline plasma PCT concentration. PCT, procalcitonin.

In a crude Kaplan-Meyer analysis we found that the cumulative incidence of all-cause mortality in the study population increased by quartile of baseline plasma PCT concentration (Figure 1). Further, we examined the association between PCT quartiles, total mortality, cancer mortality, CV mortality and mortality due to other causes in a Cox proportional hazards model corrected for age and sex (Table 2). We analyzed the entire cohort as a whole, and men and women separately. We found significant positive relationships between PCT and the risk of total mortality, cancer and CV mortality in the entire study population. The association between PCT and total mortality remained valid when men and women were analyzed separately. However, PCT predicted cancer mortality only in men and CV mortality only in women. PCT did not predict death by causes other than CV disease and cancer in any of the considered groups.

In order to assess whether PCT is independently related to mortality risk we conducted a Cox regression analysis further adjusted for BMI, smoking, hypertension, LDL, HDL, TG, diabetes mellitus and cystatin C (Table 3). Cystatin C was included in the adjusted model, as we have found a significant correlation between PCT quartiles and cystatin C in our cohort (Table 1; Spearman correlation coefficient 0.304, *P <*0.001). These results are in accordance with previous data showing that renal function influences plasma PCT concentrations [24]. The correlations between PCT, total mortality and cancer mortality in the entire cohort remained significant after adjustment. However, these relationships seem to be supported by a strong correlation between PCT and cancer mortality in men. Men having baseline PCT concentrations within the fourth quartile had a 2.37 (95% CI (1.36 to 4.14)) times higher risk of dying from cancer compared to men within the first quartile. The correlations between PCT, total mortality and CV mortality in women completely lost statistical significance after risk factor adjustment.

**Table 3:** Correlation between PCT quartiles and mortality adjusted for age, sex, hypertension, diabetes, plasma lipids, renal function, BMI and smoking

**Subjects**	**Outcome**	**Number of events**^**a**^	**Q2 versus Q1**	**Q3 versus Q1**	**Q4 versus Q1**	***P *for linear trend**
			**HR (95% CI)**	**HR (95% CI)**	**HR (95% CI)**	
All subjects	All-cause mortality	8.39	1.04 (0.76 to 1.42)	1.22 (0.90 to 1.65)	1.52 (1.13 to 2.06)	0.002**
	Cancer mortality	3.88	1.11 (0.70 to 1.74)	1.56 (1.01 to 2.41)	1.78 (1.13 to 2.77)	0.004**
	CVD mortality	2.34	1.18 (0.64 to 2.19)	1.01 (0.55 to 1.85)	1.42 (0.79 to 2.57)	0.266
	Mortality from causes other than cancer and CVD	2.16	0.90 (0.49 to 1.66)	1.07 (0.59 to1.93)	1.47 (0.82 to 2.63)	0.126
Men	All-cause mortality	11.14	0.99 (0.67 to 1.47)	0.96 (0.66 to 1.40)	1.52 (1.07 to 2.16)	0.024*
	Cancer mortality	4.92	1.71 (0.95 to 3.07)	1.38 (0.76 to 2.50)	2.37 (1.36 to 4.14)	0.006**
	CVD mortality	3.13	0.66 (0.30 to 1.45)	0.69 (0.34 to 1.39)	1.18 (0.62 to 2.25)	0.539
	Mortality from causes other than cancer and CVD	3.09	0.71 (0.33 to 1.53)	0.91 (0.46 to 1.78)	1.28 (0.67 to 2.45)	0.428
Women	All-cause mortality	6.30	1.29 (0.76 to 2.20)	1.37 (0.79 to 2.38)	1.61 (0.94 to 2.75)	0.081
	Cancer mortality	3.13	1.43 (0.72 to 2.83)	1.17 (0.55 to 2.48)	1.57 (0.76 to 3.23)	0.352
	CVD mortality	1.74	0.86 (0.27 to 2.97)	1.38 (0.43 to 4.44)	1.47 (0.47 to 4.62)	0.245
	Mortality from causes other than cancer and CVD	1.46	1.50 (0.47 to 4.77)	1.93 (0.59 to 6.28)	1.90 (0.58 to 6.16)	0.281

Cox regression analysis adjusted for age, sex, hypertension, diabetes, LDL, HDL, TG, renal function, BMI and smoking. ^**a**^Expressed per 1,000 person years. BMI, body mass index; CI, confidence interval; CVD, cardiovascular disease; HDL, high-density lipoprotein; HR, hazard ratio; LDL, low-density lipoprotein; PCT, procalcitonin; Q, quartile; TG, triglycerides. *P <0.05, **P <0.01.

We have previously described a high degree of co-variation between PCT and hsCRP in healthy individuals, which restricts the value of PCT as an independent predictor of CVD [18]. Here, we explored the value of PCT and hsCRP as independent mortality predictors in Cox regression analyses adjusted for traditional risk factors and mutually adjusted for each other (Table 4). PCT maintained significant associations with total mortality and cancer mortality in the whole population and in men, independently of hsCRP. In turn, hsCRP was independently associated with CV mortality, which is in line with our previous results [18]. We found no relationship between hsCRP, all-cause and cancer death in this model. Neither biomarker was associated with mortality in women.

**Table 4:** Comparative analysis of the associations between PCT, hsCRP and incident mortality

**Subjects**	**Outcome**	**PCT**		**hsCRP**	
		**HR (95% CI)**^**a**^	***P***	**HR (95% CI)**^**a**^	***P***
All subjects	All-cause mortality	1.15 (1.04 to 1.26)	0.006**	1.08 (0.98 to 1.19)	0.100
	Cancer mortality	1.23 (1.06 to 1.41)	0.005**	1.02 (0.89 to 1.16)	0.788
	CVD mortality	1.08 (0.89 to 1.30)	0.425	1.24 (1.03 to 1.49)	0.025*
	Mortality from causes other than cancer and CVD	1.15 (0.95 to 1.39)	0.161	1.07 (0.89 to 1.28)	0.483
Men	All-cause mortality	1.13 (1.00 to 1.27)	0.047*	1.09 (0.97 to 1.23)	0.156
	Cancer mortality	1.29 (1.08 to 1.54)	0.006**	0.96 (0.80 to 1.14)	0.632
	CVD mortality	1.04 (0.83 to 1.30)	0.741	1.30 (1.01 to 1.66)	0.037*
	Mortality from causes other than cancer and CVD	1.07 (0.86 to 1.34)	0.556	1.16 (0.92 to 1.47)	0.220
Women	All-cause mortality	1.13 (0.97 to 1.32)	0.106	1.07 (0.92 to 1.24)	0.362
	Cancer mortality	1.09 (0.88 to 1.35)	0.430	1.13 (0.92 to 1.38)	0.261
	CVD mortality	1.19 (0.87 to 1.62)	0.286	1.13 (0.84 to 1.51)	0.433
	Mortality from causes other than cancer and CVD	1.21 (0.87 to 1.67)	0.251	0.92 (0.68 to 1.23)	0.568

Cox regression analysis adjusted for age, sex, hypertension, diabetes, LDL, HDL, TG, renal function, BMI and smoking. ^**a**^The HR values are expressed as mean HR per quartile of the respective biomarker. BMI, body mass index; CI, confidence interval; CVD, cardiovascular disease; HDL, high-density lipoprotein; HR, hazard ratio; hsCRP, high sensitivity C-reactive protein; LDL, low-density lipoprotein; PCT, procalcitonin; TG, triglycerides. *P <0.05, **P <0.01.

### PCT and the incidence of cancer

We further explored whether PCT is associated with the incidence of a particular type of cancer. There were 601 cases of incident cancer during follow-up, with an equal total distribution between men and women (Table 5). The most dominant cancer subtypes were prostate cancer in men and breast cancer in women. Only cancer subtypes with more than 20 incident events in the entire population were considered for the statistical analysis. In a Cox proportional hazards model corrected for age and sex, PCT was significantly associated with the incidence of colon cancer in men (Table 5). We found no relationship between PCT and cancer incidence in women. BMI, smoking and hsCRP were previously shown to be associated with the incidence of cancer [6,25,26] and renal function influences the concentration of plasma PCT [24]. After additional adjustment for BMI, smoking, hsCRP and cystatin C the association between PCT and colon cancer incidence in men remained statistically significant (HR per 1 SD PCT increment (95% CI) = 1.45 (1.03 to 2.04); *P* = 0.034).

**Table 5:** PCT and the incidence of cancer

**Cancer subtype**	**All subjects**			**Men**			**Women**		
	**N**	**HR**^**a**^	***P***^**b**^	**N**	**HR**^**a**^	***P***^**c**^	**N**	**HR**^**a**^	***P***^**c**^
		**(95% CI)**			**(95% CI)**			**(95% CI)**	
Total cancer incidence	520	1.02 (0.93 to 1.12)	0.611	257	1.08 (0.96 to 1.21)	0.184	263	0.97 (0.86 to 1.11)	0.705
Colon	36	1.18 (0.90 to 1.55)	0.238	19	1.49 (1.13 to 1.95)	0.005**	17	0.59 (0.31 to 1.13)	0.108
Rectum	34	1.16 (0.86 to 1.57)	0.343	15	1.14 (0.71 to 1.83)	0.583	19	1.15 (0.79 to 1.67)	0.469
Lung and bronchi	43	0.95 (0.68 to 1.33)	0.764	23	0.95 (0.62 to 1.46)	0.819	20	0.96 (0.59 to 1.54)	0.864
Urinary tract	36	0.87 (0.59 to 1.27)	0.467	22	0.86 (0.55 to 1.35)	0.515	14	0.93 (0.52 to1.68)	0.815
Malignant melanoma	22	1.00 (0.64 to 1.57)	0.999	14	1.17 (0.74 to 1.87)	0.499	8	0.64 (0.26 to 1.62)	0.348
Skin (excluding melanoma)	29	0.88 (0.65 to 1.45)	0.879	19	1.12 (0.74 to 1.70)	0.600	10	0.59 (0.25 to 1.38)	0.225
Prostate				130	1.02 (0.86 to 1.21)	0.841			
Breast							122	1.00 (0.83 to 1.20)	0.998

^**a**^Expressed per 1 SD increment. Cox regression analysis adjusted for age and sex^**b**^ or age^**c**^. CI, confidence interval; HR, hazard ratio; PCT, procalcitonin. **P <0.01.

### PCT as clinical predictor of morbidity and mortality in men

As PCT is independently correlated with morbidity and mortality in men, we further investigated whether PCT may represent a robust clinical predictor of total mortality, cancer mortality and colon cancer incidence in men. The areas under the ROC curve (AUC) were 0.573 (95% CI (0.532 to 0.614)) for total mortality, 0.586 (95% CI (0.531 to 0.642)) for cancer mortality and 0.705 (95% CI (0.619 to 0.791)) for the incidence of colon cancer. Due to the modest AUC values and the low incidence of colon cancer in the male population in our study, we were not able to establish clear cut-off values with good sensitivity and specificity for PCT as a morbidity and mortality predictor in men.

## Discussion

Our data reveal a previously undisclosed relationship between plasma PCT levels in apparently healthy individuals and the risk of future mortality. PCT presented gender-specific association patterns. In men, PCT was correlated with the incidence of total mortality and cancer mortality, independently of previously disclosed risk factors for cancer and CVD. Additionally, we found that higher PCT levels were related to increased incidence of colon cancer. However, the subsequent ROC analyses suggested that PCT probably has limited value as a clinical predictor of cancer morbidity and mortality in apparently healthy men. In women, PCT was associated with the risk of total mortality and CV mortality, but the associations lost statistical significance after taking possible confounders into account. In a comparative analysis adjusted for possible confounders and each other, PCT was associated with all-cause and cancer mortality and hsCRP with CV mortality in the entire study population and in men analyzed separately. Neither biomarker predicted mortality in women.

The value of PCT as a biomarker in certain types of cancer has previously been investigated. PCT has been shown to have both a diagnostic and a prognostic value in thyroid cancer, as it is mainly produced in the neuroendocrine C cells of the thyroid gland [27]. In patients with solid tumors who developed febrile neutropenia during chemotherapy, elevated PCT was associated with treatment failure, death and sepsis [28]. A recently published study demonstrated similar results in non-neutropenic cancer patients [29].

All the studies performed so far on PCT and cancer included patients with already diagnosed disease. We are the first to report a link between higher plasma PCT levels in individuals with no previous history of cancer and increased risk of cancer mortality. The biological importance of PCT *in vivo* at low concentrations in healthy individuals has so far been unexplored. Chronic inflammation plays a pathogenic role in tumor initiation and progression [2] and hsCRP has previously been shown to predict total cancer incidence and the incidence of certain types of cancer in healthy individuals [6]. It has been shown that all tissues, including the ubiquitously present adipose tissue, are able to produce PCT under the stimulation of inflammatory mediators and bacterial products [15]. However, the specific stimuli of PCT secretion in healthy individuals other than bacterial endotoxin are currently unknown. PCT presented distinct association patterns with cancer and CV disease compared to hsCRP, suggesting that plasma PCT might reflect specific ongoing subclinical inflammatory processes rather than being a global marker of systemic inflammation, such as hsCRP.

Approximately 15% of malignancies worldwide are believed to be related to chronic infections [30] through mechanisms involving chronic local inflammation leading to DNA damage and mutagenesis [31]. In sepsis PCT plays a dual role as a diagnostic and prognostic biomarker and as a disease mediator, due to its pro-inflammatory and immunosuppressive properties [15]. In experimental models of sepsis, administration of antibodies against PCT markedly increased survival of hamsters and pigs [32] and PCT has been proposed as a therapeutic target in sepsis patients [15]. Clinically relevant doses of PCT, similar to those achieved in sepsis, induced the secretion of the pro-inflammatory cytokines TNFα, IL-1β and IL-6 in whole human blood cells *in vitro*[16]. In turn, TNFα is a strong stimulator of PCT production and IL-1β and IL-6 have been linked with elevated PCT levels, thus creating potential pro-inflammatory positive feedback loops [15,33,34]. TNFα, IL-6 and IL-17 are important links between chronic inflammation and tumor development [35]. Of note, although a monocyte chemoattractant in itself, PCT suppresses chemokine-induced human monocyte migration [17] and inhibits human neutrophil mobility *in vitro*[16]. Hypothetically, the high-responders, who have increased PCT levels under homeostatic conditions, may produce higher and more sustained amounts of PCT under bacterial and inflammatory stimulation. Subsequently, PCT may amplify the pro-carcinogenic inflammatory response and impair the anti-tumor immune mechanisms, thus acting as a disease mediator in cancer.

Although inflammation is thought to play an important role in the pathogenesis of cancer, not all types of cancer manifest the same pattern of association with inflammatory markers and mediators. CRP was previously shown to predict the incidence of cancer of any type, lung and colorectal cancer, but not breast or prostate cancer [6]. Here, we demonstrate a significant independent association between PCT levels in healthy individuals and the incidence of colon cancer. This relationship was only valid in men, who generally have higher plasma PCT concentrations than women [18]. Among the different types of cancer, colorectal cancer has one of the strongest documented associations with inflammation [36]. Patients suffering from local chronic inflammatory conditions such as Crohn’s disease and ulcerative colitis run a markedly increased risk of developing colorectal cancer, depending on the duration and the extent of the disease [36]. The PCT stimulators TNFα and IL-1β are actively involved in the pathogenesis of colon cancer and have been proposed as therapeutic targets for anti-cancer therapy [37].

The main limitations of our study are related to the relatively low numbers of subjects within each individual cancer subtype group, except for prostate and breast cancer. Based on a population of 3,322 individuals from the general population, our study was underpowered for detecting relationships between PCT and individual cancer subtypes. The lack of association between PCT and the other considered tumor subtypes does not rule out with certainty the hypothesis that such relationships might exist. Larger and more specifically powered studies are needed in order to detect whether particular cancer subtypes, potentially those related to infectious and inflammatory conditions, drive the demonstrated association between PCT and cancer mortality. PCT measurements in healthy men using the ProCa-S assay are robust, as most PCT values in this population lie above the reported functional sensitivity of the assay of 17 pg/mL. However, exact determinations of plasma PCT concentrations in healthy women are less reliable due to the generally lower PCT levels in this group. Subsequently, we cannot exclude with certainty that this limitation of the assay might have contributed to the observed lack of correlation between plasma PCT and mortality in women.

## Conclusions

In conclusion, we demonstrate for the first time in a large prospective study that baseline PCT levels are independently associated with all-cause and cancer mortality in apparently healthy men with no previous history of cancer or CV disease. Additionally, we found a significant relationship between PCT and the incidence of colon cancer in men. PCT and hsCRP seem to have complementary value as mortality risk predictors. In comparative analyses mutually adjusted for each other, PCT was independently associated with all-cause and cancer death and hsCRP was correlated with CV death in men, but not in women. The lack of independent association between these biomarkers of systemic inflammation and mortality in women after accounting for traditional risk factors may have important implications for the design and interpretation of future biomarker studies on cardiovascular disease and cancer. PCT levels have previously been shown to be associated with prognosis in sepsis, cancer and CV disease patients. Our study extends these findings in healthy individuals and warrants further investigation into the mechanisms linking PCT with the risk of cancer.

## Abbreviations

AUC: Area under the curve; BMI: Body mass index; CI: Confidence interval; hsCRP: High sensitivity C-reactive protein; CV: Cardiovascular; HDL-C: High-density lipoprotein cholesterol; HR: Hazard ratio; IL: Interleukin; ICD: International Classification of Diseases; LDL-C: low-density lipoprotein cholesterol; MDC: Malmö Diet and Cancer Study; PCT: Procalcitonin; ROC: Receiver operating characteristic; TG: Triglycerides; TNFα: Tumor necrosis factor α.

## Competing interests

The authors declared that they have no competing interest.

## Authors’ contributions

OSC participated in the statistical analysis, data interpretation and manuscript drafting. JM, GE and BH helped with data analysis and interpretation and have critically revised the manuscript for intellectual content. OM and AS conceived and designed the study and participated in the statistical analysis, data interpretation and manuscript drafting. All authors have read and approved the final manuscript.

## Pre-publication history

The pre-publication history for this paper can be accessed here:

http://www.biomedcentral.com/1741-7015/11/180/prepub

## Supplementary Material

Additional file 1 **Table S1.** Baseline characteristics of the excluded participants. **Table S2.** Baseline characteristics of the study population by cause of death. Click here for file
