# Supplementary material for: Plasma procalcitonin is associated with all-cause and cancer mortality in apparently healthy men: a prospective population-based study
Source: BMC Med. 2013 Aug 13;11:180. doi: 10.1186/1741-7015-11-180 (PMC3765625; doi:10.1186/1741-7015-11-180)
Supplement: Additional file 1 — Table S1. Baseline characteristics of the excluded participants. Table S2. Baseline characteristics of the study population by cause of death. [file 1741-7015-11-180-S1.pdf]

## Additional data file

### Additional table 1. Baseline characteristics of the excluded participants†

| Characteristic | Population | Men | Women |
|---|---|---|---|
| **Number of participants (%)** | 2772 | 1104 (40) | 1668 (60) |
| **Age, mean (SD), y** | 57 (6) | 57 (6) | 57 (6) |
| **Blood pressure, mean (SD), mm Hg** | | | |
| **Systolic** | 141 (19) | 143 (19) | 139 (19) |
| **Diastolic** | 87 (10) | 90 (10) | 85 (9) |
| **Hypertension, No (%)** | 1768 (63.8) | 783 (70.9) | 985 (59.1) |
| **Lipids, mean (SD), mmol/L** | | | |
| **LDL-C** | 4.16 (0.99) | 4.11 (0.88) | 4.19 (1.05) |
| **HDL-C** | 1.37 (0.38) | 1.18 (0.32) | 1.48 (0.37) |
| **Body mass index, mean (SD), kg/m$^2$** | 26.0 (4.0) | 26.6 (3.6) | 25.6 (4.3) |
| **Diabetes mellitus, No. (%)** | 214 (7.7) | 118 (10.7) | 96 (5.8) |
| **Current smoking, No. (%)** | 754 (27.2) | 299 (27.1) | 455 (27.3) |
| **Cystatin C, mean (SD), mg/L** | 0.79 (0.17) | 0.82 (0.18) | 0.78 (0.16) |
| **hsCRP, median (IQR), mg/L** | 0.15 (0.08-0.30) | 0.16 (0.09-0.31) | 0.14 (0.07-0.29) |
| **PCT, median (IQR), pg/mL** | 17 (13-22) | 21 (16-27) | 15 (12-19) |

† Subjects from the CV arm of the Malmö Diet and Cancer cohort that were excluded from the present study. Abbreviations: CRP, C-reactive protein; LDL-C, low-density lipoprotein cholesterol; HDL-C, high-density lipoprotein cholesterol; PCT, procalcitonin.

**Additional table 2. Baseline characteristics of the study population by cause of death**

| Characteristic | Alive | CV death | Cancer death | Death by other causes |
|---|---|---|---|---|
| **Number of participants (%)** | 2888 | 121 | 201 | 112 |
| **Age, mean (SD), y** | 57 (6) | 62 (4) | 60 (6) | 61 (5) |
| **Blood pressure, mean (SD), mm Hg** | | | | |
| **Systolic** | 141 (18) | 154 (21) | 145 (20) | 146 (20) |
| **Diastolic** | 87 (9) | 92 (10) | 88 (9) | 88 (10) |
| **Hypertension, No (%)** | 1798 (62.2) | 103 (85.1) | 134 (66.7) | 83 (74.8) |
| **Lipids, mean (SD), mmol/L** | | | | |
| **LDL-C** | 4.15 (0.97) | 4.30 (1.04) | 4.19 (1.02) | 4.25 (1.07) |
| **HDL-C** | 1.40 (0.37) | 1.27 (0.34) | 1.30 (0.36) | 1.36 (0.39) |
| **TG** | 1.29 (0.62) | 1.45 (0.67) | 1.41 (0.65) | 1.36 (0.71) |
| **Body mass index, mean (SD), kg/m$^2$** | 25.7 (3.9) | 26.8 (3.9) | 26.0 (3.6) | 24.8 (4.4) |
| **Diabetes mellitus, No. (%)** | 209 (7.2) | 29 (24.0) | 18 (9.0) | 16 (14.4) |
| **Current smoking, No. (%)** | 691 (23.9) | 47 (38.8) | 80 (39.8) | 43 (38.7) |
| **Cystatin C, mean (SD), mg/L** | 0.76 (0.13) | 0.87 (0.17) | 0.78 (0.13) | 0.84 (0.30) |
| **hsCRP, median (IQR), mg/L** | 0.12 (0.06-0.26) | 0.27 (0.08-0.50) | 0.17 (0.07-0.34) | 0.17 (0.06-0.36) |
| **PCT, median (IQR), pg/mL** | 16 (13-20) | 19 (15-23) | 18 (14-23) | 18 (14-19) |
| **Men** | 18 (15-22) | 20 (16-25) | 20 (17-26) | 19 (15-26) |
| **Women** | 14 (12-18) | 18 (14-22) | 15 (13-19) | 17 (13-22) |

Abbreviations: CRP, C-reactive protein; LDL-C, low-density lipoprotein cholesterol; HDL-C, high-density lipoprotein cholesterol; PCT, procalcitonin.
